# Malaysian Stakeholder Perspectives on Suicide-Related Reporting: Findings From Focus Group Discussions

**DOI:** 10.3389/fpsyg.2021.673287

**Published:** 2021-05-17

**Authors:** Yin Ping Ng, Kai Shuen Pheh, Ravivarma Rao Panirselvam, Wen Li Chan, Joanne Bee Yin Lim, Jane Tze Yn Lim, Kok Keong Leong, Sara Bartlett, Kok Wai Tay, Lai Fong Chan

**Affiliations:** ^1^Pantai Hospital Penang, Bayan Lepas, Malaysia; ^2^Department of Psychology and Counselling, Faculty of Arts and Social Science, Universiti Tunku Abdul Rahman, Kampar, Malaysia; ^3^Department of Psychiatry, Miri Hospital, Miri, Malaysia; ^4^Nottingham University Business School, University of Nottingham Malaysia Campus, Semenyih, Malaysia; ^5^School of Media, Languages & Cultures, Faculty of Arts and Social Sciences, University of Nottingham Malaysia Campus, Semenyih, Malaysia; ^6^Department of Psychiatry, Faculty of Medicine, National University of Malaysia, Kuala Lumpur, Malaysia; ^7^Hospital Tuanku Ja’afar Seremban, Seremban, Seremban; ^8^Everymind, Newcastle, NSW, Australia

**Keywords:** suicide prevention, focus group discussion (FGD), safe reporting, media, stakeholders, suicide, media guidelines

## Abstract

Media guidelines on safe suicide-related reporting are within the suicide prevention armamentarium. However, implementation issues beleaguer real-world practice. This study evaluated the perspectives of the Malaysian media community, persons with lived experience of suicidal behavior (PLE), and mental health professionals (MHP) on suicide-related reporting in terms of the impact, strategies, challenges, and the implementation of guidelines on safe reporting. Three focus group discussions of purposively sampled Malaysian media practitioners (*n* = 8), PLE (*n* = 6), and MHP (*n* = 7) were audio-recorded, transcribed, coded and thematically analyzed. Inclusion criteria were: English fluency, no clinical depression or suicidal ideation (current), no recent previous suicide attempts or suicide bereavement. Three major themes emerged: (1) Unsafe Reporting; (2) Impact; and (3) Safe Reporting. Most described current reporting as unsafe by being potentially triggering to media users and may contribute to contagion effect. Positive impacts identified included raised awareness toward suicide and its prevention. Unsafe reporting was attributed to inadequate awareness, knowledge, and guidance, lack of empathy and accountability, job-related factors, popularity-seeking, lack of monitoring and governance, and information source(s) with unsafe content. Majority agreed on how suicide stories should be framed to produce a safe report. The media community diverged on how detailed a suicide story should be. Safe reporting challenges included difficulties in balancing beneficial versus harmful details, social media ubiquity and its citizen reporters. Participants suggested these safe reporting strategies: stakeholder engagement, educational approaches, improving governance and surveillance, and guidelines revision. Most acknowledged the relevance of guidelines but were unaware of the existence of local guidelines. Implementation challenges included the dilemma in balancing media industry needs vis-à-vis safe reporting requirements, stakeholder engagement difficulties and social media regulation. There is poor awareness regarding safe suicide-related reporting across all groups. PLE and MHP were negatively impacted by current unsafe messaging which aggravated trauma and grief reactions. Postvention support gaps for mental health professionals were highlighted. Safe reporting promotion strategies should include stakeholder engagement to increase awareness on minimizing Werther and maximizing Papageno effects. Strategic re-examination and dissemination of local media guidelines to address new media issues, and effective surveillance mechanisms, are crucial in sustainable improvement of safe reporting practices.

## Introduction

The reporting and portrayal of suicide in the media has significant societal and public health implications (Ng et al., 2021). The risk of suicide contagion from media reports of suicide, more commonly known as copy-cat suicide, or the Werther effect, has been documented and debated in over 150 published studies to date (Niederkrotenthaler et al., 2020). More recently, suicide preventive elements of media reports related to suicide events have been described as the Papageno effect, whereby the media portrayal of how a person successfully overcomes a suicidal crisis has been associated with a reduction of suicide rates at the population level (Niederkrotenthaler et al., 2010). In view of the potentially harmful and protective effects of suicide-reporting, media guidelines have been developed based on the World Health Organization (WHO)’s recommendations as a reference point for safe and responsible reporting of suicide (Beautrais et al., 2008). Such guidelines have been implemented worldwide with varying degrees of success in terms of acceptance and enforcement (Bohanna and Wang, 2012). In Malaysia, guidelines for media reporting on suicide were developed in 2004 by the Ministry of Health with the input of mental health practitioners and representatives from the media (Malaysia Ministry of Health, 2004). However, more than a decade later, suicide reporting practices in Malaysia remain largely incongruent to recommendations in the guidelines (Johari et al., 2017; Chan et al., 2018; Victor et al., 2019).

Beyond the population effects of media reporting on suicide rates, the level and quality of stakeholders’ collaborative engagement on the ground is crucial for successful and sustainable implementation of media guidelines for suicide prevention (Cheng et al., 2014). Media guidelines on safe suicide reporting should ideally be informed by the expertise and knowledge of parties who have to deal with the ramifications of inappropriate coverage (Tully and Elsaka, 2004; Norris et al., 2006; Bohanna and Wang, 2012; Duncan and Luce, 2020). On this front, studies have explored journalists’ experiences and perspectives on suicide reporting. In (Cheng et al., 2014) ’s study, media practitioners provided the following factors as rationale for the intensive suicide-reporting in Hong Kong: (i) economic competitiveness, (ii) audience appetite, and (iii) the media’s perceived role as the voice of public consciousnesses in relation to social issues. In New Zealand, in addition to a similar ethos of “promoting the public good”, (Collings and Kemp, 2010)’s qualitative study among media practitioners provided insights on other journalistic experiences such as media framing of suicide, professional practice and restricted reporting, and how professionalism buffered the psychological distress of suicide-reporting via emotional distancing. What is less understood is the impact on the suicide-bereaved of reporting and editorial decisions of suicide-related events in the media.

Existing literature on media reporting of suicide highlighted the diversity of suicide bereaved experiences in terms of what is constituted as acceptable in responsible media suicide reporting (Chapple et al., 2013)’s UK study found a difference in emphasis between media guidance on suicide reporting, and the perspectives and needs of persons bereaved by suicide. The delicate balance between preventing future suicides and protecting the interests of those bereaved by suicide was acknowledged by Gregory et al. (2020). A suicide prevention-focused style of reporting was highlighted as potentially positive in terms of the impact on suicide bereavement (Skehan et al., 2013).

There is a paucity of published data on the qualitative experiences of vulnerable populations such as people with lived experience of suicidal behavior with regards to the impact of suicide-reporting and the role of media guidelines. Mental health professionals are also significant, albeit under-studied, stakeholders in the area of suicide-reporting and the content of media guidelines. Notwithstanding their professional role as suicide prevention clinicians, mental health professionals are not immune to the negative impact of suicide-reporting in view of their high exposure to client suicide (Seguin et al., 2014).

Knowledge gaps exist with regard to the need for insights from key understudied stakeholders on the reporting and portrayal of suicide-related events in the media. This has important implications on the ecosystem of stakeholder engagement for the strategic implementation of safe suicide-reporting media guidelines. Therefore, the objective of this study is to explore the perspectives of culturally diverse Malaysian media practitioners, persons with lived experience of suicidal behavior and mental health professionals on the current state of suicide reporting, challenges and strategies for safe reporting, and media guideline use.

## Materials and Methods

### Study Design

This study employed thematic analysis (Braun and Clarke, 2006) which used an inductive approach. The study was conducted in November 2018 within a media safe-messaging advocacy event at a patient support group organization venue.

### Recruitment and Sampling

Participants were recruited prior to the media safe-messaging advocacy event. Recruitment by purposive and snowball sampling was communicated through networks of the research team via email, social media (e.g., Facebook, Twitter, and Instagram), instant messaging applications (e.g., WhatsApp), organizational/institutional email listserv/mailing lists, telephone calls or face-to-face meetings. Examples of these networks included non-governmental organizations (e.g., Malaysian Psychiatric Association, MPA), patient advocacy groups (e.g., Mental Illness Awareness and Support Association, MIASA) and social enterprise networks (e.g., Thoughtfull, *Laman Minda*). The inclusion criteria were: (i) aged 18 years old or older, (ii) either a media practitioner or media student, mental health professional, or person with lived experience of suicidal behavior [either personal or significant other)], (iii) sufficiently proficient in the English language, (iv) not clinically depressed based on a Patient Health Questionnaire-9 (PHQ-9) score of less than 10 (Kroenke et al., 2001), (v) had not had active suicidal thoughts or plans in the 2 weeks prior to the focus group discussion (FGD), any suicide attempt in the 6 months prior to the FGD or been bereaved by suicide in the 6 months prior to the FGD (Skehan et al., 2013). Exclusion criteria were non-fulfillment of any of the inclusion criteria. Help-seeking resources (i.e., crisis lines, information on accessibility and facilitation of referral to mental health services) were made available to every person who gave informed consent for the study including those who were excluded from the FGD. Study participants were given assurance that if they felt uncomfortable or the need to leave the session at any point, they could indicate so by raising their hand. The assistant moderator was on standby to attend to such needs. After each FGD, each participant was given a Post-FGD Screening Questionnaire (PHQ items-2 and 9) to complete. Provisions were made for participants who expressed any emotional distress or screened positive from the questionnaire, to be provided with supportive counseling (i.e., listening to their concerns and validating their emotions), help-seeking resources and facilitation of referral to mental health services.

There was no financial incentive provided for participation in the study.

### Data Collection and Data Analysis

We collected data using focus group discussions (FGDs). Three focus groups were formed, representing the three stakeholder groups in the study, namely, persons with lived experience of suicidal behavior (either personal or significant other) (PLE), media practitioners, and mental health professionals (MHP). Each focus group consisted of 6–8 participants which is within the recommended sample size of 5–13 per focus group according to Matthews and Ross (2010).

All three FGDs were carried out simultaneously in different rooms and conducted in the English language. Each FGD was moderated by one of the researchers and audio-recorded, while a second researcher or trained research assistant took field notes. All 3 moderators reached consensus on standardizing the interview moderation prior to the FGD. All 3 moderators are experienced clinical psychiatrists (average of 6 years). Two of the moderators (CLF and RRP) received specific training in FGD from one of the moderators (NYP) with a Master of Science in Health research (MScHR) that included conducting FGDs in qualitative research. The FGDs were conducted with reference to a semi-structured interview guide (Table 1), with the moderator utilizing semi-structured, open-ended interview questions to guide the discussion. Subsequent questions that followed were directed by participant responses, with prompts from the moderator until saturation point was reached. The duration of each FGD was approximately 2 h. All audio recordings were transcribed verbatim.

**TABLE 1:** Semi-structured interview guide.

**Semi-Structured Interview Guide**
• Some of you may have either read or reported on suicide-related content in the media. How has the experience affected you?
• What is your opinion on how an article with suicide-related content is (or should be) portrayed in the media?
• With regards to existing media reporting guidelines on suicide-related content, what is your opinion of guidelines for suicide-related content in the media?
• How can we improve in reporting suicide-related content?

The transcriptions were thematically analyzed by at least three different researchers. Each set of transcripts and field notes were repeatedly examined by each researcher, and the findings were discussed to achieve a consensus, to ensure objective interpretation of participant responses. We created codes and a coding template which contained code definitions to organize the raw data. These codes were later collated to search for emerging patterns of meaning (themes), reviewed to redefine the main overarching themes, and finally triangulated with the observation notes to enhance the findings.

This study was reviewed and approved by the Universiti Tunku Abdul Rahman Scientific and Ethical Review Committee (U/SERC/119/2018).

## Results

### Profile of Participants

We recruited 6 PLEs, 8 media practitioners and 7 MHPs. Two people who gave informed consent were excluded from study participation - one was unable to attend the FGD due to an upper-respiratory tract infection, and the other person screened positive for suicidal thoughts in the 2 weeks prior to the FGD based on item-9 of the PHQ-9. In terms of general group dynamics, there was no dominance of the FGDs by any particular study participant. One participant was given supportive counseling following the FGD by an assigned assistant researcher who is a trained clinical psychologist; a suicide risk assessment with safety planning and facilitation to the necessary mental health resources and supports was also carried out. Participant characteristics are summarized in Table 2. All participants had encountered suicide-related stories in the media.

**TABLE 2:** Participant characteristics.

**Focus group**	**Participant**	**Lived experience (LE)**		**Category**
		**(Yes = Y, No = N)**	**If Y, type of LE**	
**Media community (*N* = 8)**	D	N	Not applicable	Freelance journalist (trauma)
	J	N	Not applicable	Media student
	G	N	Not applicable	Journalist and producer
	C	Y	Suicidal behavior (SO)	Media management
	B	N	Not applicable	Media student
	H	Y	Suicidal behavior (SO)	Journalist (radio)
	E	Y	Suicide-bereaved	Journalist (print)
	A	Y	Suicide-bereaved	Media student
**Persons with lived experience (*N* = 6)**	M	Y	Suicide-bereaved	Administrator
	Mg	Y	Suicidal behavior (SO)	Pensioner
	KC	Y	Suicide-bereaved, suicidal behavior (P, SO)	Communicator
	LL	Y	Suicidal behavior (P, SO)	Student
	N	Y	Suicidal behavior (P, SO)	Marketer
	Bt	Y	Suicide-bereaved, Suicidal behavior (SO)	Educator
**Mental health professionals (*N* = 7)**	I	Y	Suicide bereaved (C)	Psychiatrist
	W	Y	Suicide bereaved (C)	Psychiatrist
	J	N	Not applicable	Psychiatrist
	S	Y	Suicidal behavior (C)	Psychiatrist
	A	Y	Suicide bereaved (C)	Psychiatrist
	R	Y	Suicide bereaved (C)	Psychiatrist
	Z	Y	Suicidal behavior (C)	Psychiatrist

*Lived experience (LE) is defined as encompassing either personal experience with suicidal behavior (P), suicide bereavement, exposure to suicidal behavior of significant others (SO), and/or exposure to client suicide or client suicidal behavior in the case of MHP participants (C). The presence of lived experience is determined either directly from information obtained from participant demographic forms or via findings from the focus group interview.*

### Themes

**FIGURE 1:**
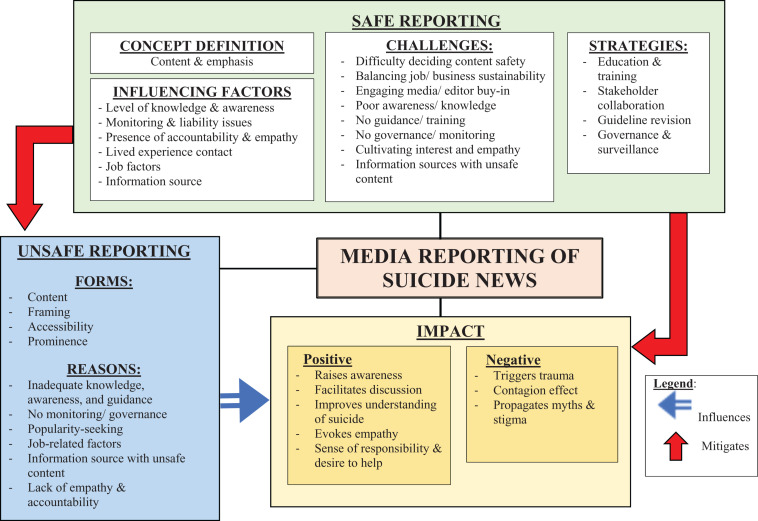
Thematic map.

Three major themes emerged from the discussion and are shown with their various subthemes in Figure 1 (Additional information on the level of consensus between all participants in the 3 FGDs can be found in the Supplementary Table).

#### Unsafe Reporting

The majority of participants agreed that the current manner in which suicide-related news is reported and published is generally unsafe and potentially harmful. Types of media discussed included print, broadcasting and digital media, especially social media. Suicide-related content on social media was recognized as a significant area of unsafe reporting. As expressed by media participant, A:

*“Social media is a completely different story because a lot of the people who are creating content on social media are not industry professionals. And a lot of these people are people’s main source of news, a lot of friends I have don’t subscribe to even digital newspapers, they get their news completely from social media sources, their friends, blogging, yeah, Facebook Live, videos.”* (A, Media).

Unsafe suicide-related material identified were in four forms: (i) content [such as detailed descriptions of methods, location and/or person(s) involved, graphic images and paucity of help-seeking resources]; (ii) framing [such as inaccurate and judgmental portrayals of the suicide or person(s) involved]; (iii) accessibility of unsafe content (such as repeated coverage of the same news and permanence of online archives), and (iv) prominence of unsafe content, in terms of front page placements and use of headlines. Although the focus of the discussions was on suicide-related reporting, it became evident that suicide-related material encountered by participants included other forms of communication beyond news reports, such as blogs, video footages by social media users and comments within each post. As such, we decided to adopt the term ‘messages or messaging’ to include such suicide-related material.

##### Reasons for unsafe reporting

Participants expressed several potential reasons behind the persistence of unsafe reporting (see Figure 1), namely inadequate knowledge, awareness and guidance related to safe reporting, lack of monitoring or governance, readership or popularity-seeking, lack of empathy and accountability, and information source(s) with unsafe content.

###### Inadequate awareness, knowledge, and guidance

The majority agreed that most people are not aware of the importance of safe reporting, nor do they realize the potential effects of unsafe suicide-related reporting.

As stated by an MHP,

*“I think not many people are aware [sic] the danger of irresponsible reporting of suicide.”* (S, MHP).

This is conceded by media participants such as E who did not realize the implications of sensationalism, nor how it would have trigger effects on someone with suicidal thoughts.

Others stated that there is also a lack of guidance specifically in the area on how to convey mental health or suicide-related information. While workshops are being conducted on defamation and libel, E added that the topic of suicide reporting is hardly prioritized.

Many were not even aware of the availability of media guidelines.

Indeed, A, a media participant, pointed out that even though there are Malaysian media guidelines available, the information did not reach media practitioners:

*“It’s just a matter of people don’t know it and it’s not enforced enough. I mean (…) how many journalists actually know what the guidelines on suicide are, how many of them would check it out (…) they’ve never been restricted from writing some like, sensationalist articles.”* (A, Media).

Others suggested that even if people knew of local media guidelines, they may be reluctant to adhere to guidelines that they perceived were developed without media participation or deemed restrictive.

*“…they don’t feel that it is actually theirs. (…) It is actually done by someone else. If they don’t feel the ownership from the guidelines, they don’t feel the responsibility to follow it, you know. (…) So they feel that we are trying to dictate what they should do or (sic) should not do.”* (R, MHP).

###### Absence of monitoring or local guidance especially with regards to posts or articles on social media

According to C, a media participant, the lack of monitoring contributes to the lack of awareness on the need for safe reporting or messaging, in addition to not being consulted on the content nor being made aware of any guidelines for suicide and mental illness reporting, even as content creators.

*“the content, the code for suicide, mental illness, is not being driven through in our decisions as content creators. (…) I don’t think there are anyone monitoring mental illnesses depiction in content.”* (C, Media).

In addition, participants highlighted that many are using social media to express themselves and it is a challenging task to keep track of posts on social media:

*“…on Facebook, a lot of graphic imagery happens to come up on Facebook Live because it’s difficult to trace in the current moment … when it happens in 2 s, … for them [social media companies] to come down on a like, deletion spree …, those blogs have already made their impact, … like twelve posts per minute, yeah, it’s a lot more difficult to restrain something so unlimited.”* (A, Media).

###### Job-related factors

Media participants cited job-related factors which at times necessitated such reporting. Firstly, suicide stories are highlighted only when they are perceived to be newsworthy –

*“I think covering a suicide follows any other news guideline which is, is it newsworthy? (…) if, it’s relevant to a wider audience, (…) because there’s an issue that we need to address, (…) what’s the bigger message for us as a society that we need to prevent this (suicide) from happening.”* (G, Media).

D conceded, stating that for celebrity suicides, particularly, the news would *“be the front page…, that will open a* (2-page) *bleed,”* which meant *“that more information and more details”* would be published.

In addition, when covering a suicide story, media participants believed that it was their job and duty to report the truth of what had taken place, and this included referring to the method of suicide.

*“As a, as a news story, (…) without giving the details, so for example, I think to say commit suicide or kill yourself is too vague to be a story, you’d have to say (methods…)”* (G, Media).

Other media participants purported that the published report ultimately depended on editorial discretion – their editors had the final say. An MHP recalled a journalist’s response when asked about the choice of language used in a published suicide report –

*“He says that my editor likes to use this kind of word. ‘I have to word this kind of word because we need to be different from other papers. In Tamil there is three main websites and stiff competition among them. So, when I (am) covering, this is one of the headline sensational news, I have to put (in this) the paper, which is not (found on) other papers.’ There is competition among them, how they report this thing.”* (W, MHP).

###### Information sources with unsafe content

Media practitioners also tended to rely on information provided by perceived authoritative sources such as the police or autopsy findings which may contain unsafe materials:

*“the authorities, the police, because we will report whatever the authorities said first.”* (D, Media).

Others from broadcast media reported similar predicaments as they relied on information from reporters as their source of news:

*“(In) radio broadcasting, we do not have our own team of reporters (…), so we rely quite heavily on newspapers. So, um, if let’s say those broadcasters who do not have the awareness or do not know about the guidelines, if the reporters really write it in a sensational way maybe, the broadcasters will just read it out.”* (H, Media).

However, some conceded that it was not necessary to include explicit details of the suicide method, “as long as it is possible to understand the story” (G, Media).

###### Lack of accountability and empathy, and popularity seeking

Participants cited lack of accountability and empathy; compounded by increasing self-interest (profit and popularity-seeking) as contributing factors to unsafe messaging in the media, including those by social media users. According to one MHP,

*“The need to be the one who posted certain news online, (…) who gets the most shares, um retweets, likes, so that somehow affect a person’s decision making in terms of deciding whether should I post this or should I not. Where your popularity matters more than the welfare of other people. So, that goes back to a person’s values.”* (Z, MHP).

#### Impact

Suicide-related media content evidently impacted each participant in one way or another. Participants identified both positive and negative impacts of suicide-related media reporting. The majority of participants cited generally negative impacts when asked to comment on the quality of current media suicide-related material. It was interesting to note that media participants (apart from media students) were quick to deny experiencing any personal adverse emotional impact from suicide-related news.

##### Negative impact

###### Triggering trauma

All participants agreed that current suicide-related reporting may potentially affect consumers in an adverse manner, particularly among people with lived experience, in that their experience of encountering suicide stories on the media either reignited traumatic memories of their own suicidal behavior or rekindled grief reactions related to suicide bereavement. One PLE participant said that the details were unimportant and irrelevant.

*“all these details (…), I think they are not important, they are not relevant. I think it really affects me because it trigger(s) all the emotions; and…all the pain and then you just feel sorrowful for the person and, sorrowful for the family.”* (Bt, PLE).

Some of the PLEs even preferred to avoid reading contents of any suicide-related article except for the headlines because,

*“…people who are writing it may not necessarily be sensitive to people who are vulnerable, like me. And for me, the most difficult time I had with these thoughts were like a decade ago and still, (pausing, getting teary) I know I can’t come, like too close to the topics.”* (N, PLE).

MHPs were equally as negatively affected by suicide-related news or posts. They were reminded of their own grief, including experiencing intrusive images and feelings of failure; toward the loss of their patients as shown by the excerpts below:

*“Seeing media reporting about your patients that you’re seeing (sic) few days before their attempt is quite traumatizing to me. It makes (sic) me think a lot, if have I done enough for them.”* (I, MHP).

Others expressed outrage and disgust at the lack of empathy and negativity related to the live recording of suicides posted on Facebook and the negative comments that accompanied the post. B (Media) claimed that she was disturbed by a thread of live tweets that seemed to ‘encourage’ a suicide attempt,

*“… if I was depressed or on the verge of suicide, if I just see how the public would respond to this, like ‘Oh, they want me to die.’ There wasn’t anyone that was tweeting stuff like ‘He should hold on a little longer”’.*

###### Contagion effect

Participants agreed that unsafe reporting of suicide-related news can potentially trigger a contagion effect. For example, one media participant (A, Media) attributed a friend’s suicide to the negative influences of suicide-related messages on social media, which led him to ‘romanticize’ suicide.

Coverage on celebrity-related suicides were especially likely to contribute to a contagion; as shown in the exerpts below:

*“but when that [death of Anthony Bourdain] happened (…), I was like (…), ‘Why, why would I still have that?’ I thought I am over it. (…) It’s been so long. I wrestled with my self-esteem for a little bit during that period because I felt like I have failed myself if I was still having these thoughts. Because of how much I relate(d) to him as a person, (…) I read up stuff online about him. (…) The more I read, the more I’m even more affected because there are so many people who love him and (pause), it just makes it even more difficult for me to deal with that incident. (…) So when I found out about the method, (pausing) it just kept playing in my head.”* (N, PLE).

###### Propagating myths and stigma

Participants believed that media suicide reports may propagate myths or inaccurate information about suicide, thereby further worsening the stigma surrounding suicides. For example, one participant (C, Media) commented that a media documentary seemed to depict the actor Robin Williams’ suicide as a form of peaceful, beautiful, and perfect death, a perfect ending; and inferred that in taking his own life he was in a sense taking control of his own life choices.

##### Positive impact

On the other hand, most participants also agreed that suicide-related news can produce positive impacts, in that reporting suicide stories can help to raise awareness on suicide matters and its severity, provide a platform to discuss about suicide and help to improve understanding related to suicide. In addition, some participants felt that suicide reporting could evoke feelings of empathy and responsibility toward preventing suicides:

*“it affects me in the sense that…we need to help people capture better…and if we have a chance to start earlier, then we can maybe have a success rate of at least preventing or helping them.”* (M, PLE).

#### Safe Reporting

The majority of participants agreed that suicide stories should be framed to embody positive messages, educate on facts related to mental health issues and suicide prevention, and include help-seeking resources. Participants from the MHP and PLE groups highlighted that there should be more article weightage on empowering and supporting people who may be seeking help and on reducing stigma, rather than focusing on the suicide act. The MHPs and PLEs also added that details or pictures which could potentially identify the decedent or their family, or pictures/suicide methods, should be avoided.

As explained by N, a PLE;

*“I don’t want to know (pause) (…) these really personal details that allow me to construct like an image of them in my head. That’s not helpful to me personally. Someone who’s going through those thoughts (pause) would understand that none of those details matter (…) because you want them to choose life.”* (N, PLE).

Participants identified several factors that could influence safe suicide reporting (see Figure 1). Having knowledge and awareness of the topic, presence of legal implications that require media guidelines to be adhered to strictly, personal values of accountability and empathy, and contact with people with lived experience of mental health issues or suicidal behavior could encourage a more empathetic and responsible manner of report writing. The latter two are illustrated by the following excerpt from media representative, A:

*“It makes you realize it’s not exactly the same as, as another story. If you’re talking about somebody who died of cancer it doesn’t spur on people to get cancer. (…) But with suicides it affects people very personally, it affects mental illness very personally. (…) When you’ve had that empathetic, um, connection with somebody [with lived experience], when you see the impact it’s had on people, it will affect the way that you write about suicide.”* (A, media).

##### Challenges to safe reporting

Although media representatives held similar views on raising awareness of suicide as an important topic, they diverged on what would be deemed safe in terms of writing about suicide-related topics/stories while at the same time keeping to journalistic commitments of ‘informing the public of the truth’ and highlighting social issues. Media participants were divided on the degree of detail especially when they needed to provide context to build the story to maintain newsworthiness, and on the use of the term ‘suicide.’

As expressed by E, a media professional – *“[Suicide] has had quite an impact, and sometimes you are at loss to what to report, because … things like triggers and stuff like that. Because we as a newspaper to be practical, you need to attract readers, and not by sensationalizing it, but even to visualize it, for example. So, we don’t put pictures of the people involved or the families because we are sensitive to them, but then you have a graph- a, a(n) illustrative image of a person standing [location], for example, which I read recently, it can also be a trigger (…) like have an effect on someone. So, it’s a little bit of a conflict, on what you can or what you cannot do.”* (E, Media).

Apart from having to delicately balance between benefit and harm in creating safe content, participants also highlighted challenges of needing to remain current and competitive in the news market, which is increasingly digital, against newer online portals who may not be aware of or governed by the standard ethics of reporting.

G of the media lamented:

*“that’s how it is, the difficulty is when traditional or professionally trained media is competing with untrained, young, new news portals. Then if they get all the digs with their click-bait headlines. Do you want to survive or you- Like how do you survive without going to that level? But you are competing with people who don’t have that understanding anyway.”* (G, Media).

Other challenges (see Figure 1) have been mentioned earlier.

##### Strategies to improve safe reporting

Participants discussed four main strategies to ensure safe reporting (see Figure 1).

###### Education and training

All participants were unanimous in advocating for better education to improve awareness and knowledge on safe reporting and its importance in relation to suicide prevention. Media participants acknowledged that there has been no specific teaching focused on how to approach issues related to mental health or suicide in the present journalism or media school syllabus. They recommended such teaching to be provided at an early stage and included in media school curricula whilst also supplemented with regular training to remind and inculcate practice in media content creators. H, a media participant added that receiving training positively changed her perspective, attitude, and approach to suicide stories:

*“How I see suicide cases, is very different before and after I’m being exposed to proper knowledge of counseling, psychology, mental health and also the guidelines. It changed my perspective after I’m being exposed to more knowledge of counseling, psychology and mental health.”* (H, Media).

###### Stakeholder collaboration

###### Engaging and collaborating with the media

Media participants such as C and D recommended engaging and training editors who usually make the final decisions on a published report. MHP participant R had the same opinion, based on observations from interactions with journalists:

*“We should target the editors rather than the reporters because most of the time, they [reporters] will just say that “oh, it’s not our fault, we just follow orders.”* (R, MHP).

Participants further provided suggestions on ways to engage media editors who were said to be often very busy and not readily available. These included suggestions by media participants to implement top-down directives via regulatory bodies on safe reporting training (G, Media), to raise awareness through the use of creative and concise video content (E, Media), to have engagement via collaborative rather than instructive means (D, Media), and to provide incentives and recognition such as awards or prizes to encourage safe reporting (KC, PLE and C, Media).

###### Collaboration with other stakeholders

Others added that safe reporting awareness should not be limited to media practitioners, but also taught to other stakeholders such as MHPs, policymakers, first responders (who often serve as information sources to the media) and the general public who are both users and contributors on social media. With improved awareness, these stakeholders can be in turn empowered to spread the awareness about the concept and importance of safe reporting to others.

*“if we can get like from multiple stakeholders explaining to them that this is the research done on it, this is what you can do, you are really going to be great partner of ours, that’s when they’ll be like, ‘Oh I can (emphasis) help other people, this is how I can positively contribute to the cause without harming someone in that sense.”* (N, PLE).

###### Revision of media guidelines

Participants highlighted that current media guidelines, although helpful, should be updated. One PLE participant, N, pointed out that the media guidelines should be revised to encompass posts and publications on digital (including social) media, including guidance for social media users on considerations in respect of posting sensitive content. Another PLE participant suggested for social media platform owners to implement automated message prompts to remind users of media guideline adherence whenever suicide-related content is posted online.

###### Governance and surveillance

Apart from education, training, stakeholder collaboration and guideline revision, a majority of participants agreed that there is a need for some degree of governance and surveillance on media publications and posts related to suicide matters. As explained by media participant, A:

*“When they write something it’s never been penalized for having, having said some things, they just don’t know the guidelines exist at all, and nobody, administrators, um, bosses- Nobody shows up and says this is wrong.”* (A, Media).

Some MHPs recommended for the setting up of a dedicated government taskforce or institute to oversee matters related to suicide prevention. One PLE added that:

*“The Ministry of Communication will play a huge role for governance (pausing), with a lot of help from the Ministry of Health in determining what needs to be filtered, what’s the proper guidelines etc.”* (N, PLE).

Some pushed for stricter regulations regarding suicide reporting, and for more punitive enforcement of media reporting guidelines:

*“for a person to change, it requires more than just knowledge. So, I think the government should be punishing those people or those newspapers that (are) (…) covering news about suicide deaths (and) not following the regulations and acts that we already have in our country. If we don’t do that, they will not adhere.”* (Z, MHP).

Others disagreed, as summarized by G, a media participant:

*“I don’t necessarily agree, and I think that this conversation goes into like, very dangerous territory, because how do you start regulating and policing individuals on social media? There’s just so many difficult gray areas. (…) It’s just so complicated. Is this something we can police or is it just a question of morals, and how do you police this and you don’t police oversexualization of music videos, you know?”* (G, Media).

## Discussion

### Unsafe Reporting

Findings from this study are congruent with earlier studies that have highlighted the preponderance of potentially harmful, suicide-descriptive, over suicide-preventive and protective elements of Malaysian media (online newspapers) in terms of content, framing and acceptability and prominence (Johari et al., 2017; Chan et al., 2018; Victor et al., 2019). Our study concurred with (Collings and Kemp, 2010)’s findings whereby media participants viewed explicit, graphic, or romanticized portrayal of suicide in the media (including methods), as unnecessary due to the risk of contagion effect. In addition, our participants highlighted the prevalence of unsafe suicide-related media messages on social media, which included online graphic images or videos, personal posts, discussions, and comments linked to suicide-related posts (which may or may not be safe). Furthermore, the comments and discussions often take a course of their own and contribute to further harmful suicide-related messages. Our participants with lived experience shared similar sentiments to those in Skehan et al. (2013)’s study in that help-seeking resources, especially on postvention services were particularly lacking.

The reasons attributed to the prevalent harmful or unsafe reporting of suicide stories in Malaysia are not dissimilar to those cited in Malaysian (Johari et al., 2017; Victor et al., 2019) and international studies (Collings and Kemp, 2010; Cheng et al., 2014; O’Brien, 2020). Poor literacy related to suicide prevention and the concept of safe suicide-related reporting (Collings and Kemp, 2010; Cheng et al., 2014; O’Brien, 2020) compounded by media organizations’ endeavor to remain commercially competitive and popular in the rise of online news portals, as well as the lack of guidance and monitoring are common factors.

In our study, the majority of participants believed they were contributing toward raising awareness on suicide issues and were unaware of the potentially negative implications related to unsafe suicide reporting. Our media participants rationalized mentioning the method of suicide without the inclusion of explicit details, as this was regarded as a professional obligation to *“report the truth”* about real-life cases of suicide in the news. This was deemed necessary for the sake of clarity in communication so that readers could understand the narrative of the story. However, there are different views regarding the degree of detail that should be included about suicide methods in the media. Others raised concerns that restrictions on suicide-related writing or content creation may impede suicide prevention work, which is similar to the findings by Collings and Kemp (2010). In addition, very few were aware of the existence of the Malaysian Ministry of Health guidelines on responsible suicide reporting (Malaysia Ministry of Health, 2004). Another area of concern is that media practitioners also relied heavily on information sources which were themselves not necessarily safe.

Media corporations focus on producing material that are deemed ‘newsworthy’ to attract consumers, capitalize on sale and remain competitive while maintaining cost effectiveness (Allern, 2002; Crane et al., 2005; O’Brien, 2020). This translates to personifying and simplifying complex stories to make them more relatable and understandable to the general public (Allern, 2002; Cheng et al., 2014). In the case of suicide reporting, articles often focus on the death event due to limited news space and tend to be produced under intense pressure of time with editors having to make quick decisions on delicate issues (Allern, 2002; Crane et al., 2005; O’Brien, 2020). The resultant outcome is overly descriptive suicide news that often oversimplify and misrepresent suicide as being monocausal in nature. Such content is potentially triggering due to the personal details that may resonate with readers who share similar attributes to the decedent; and provide sufficient details for one to potentially imitate the act (Ng et al., 2021). In addition, participants raised concerns regarding the hierarchical and competitive nature within the media industry that were thought to fuel unsafe reporting. Journalists were expected to adhere to editorial decisions based on the presumption that sensationalist headlines would increase a newspaper’s competitive edge. Cheng et al. (2014)’s study highlighted that such assumptions by the media about readers’ interest for sensationalist news conflicted with actual audience preference for less sensationalist suicide news reporting. Moreover, Frye (2005) demonstrated an inverse relationship between the level of sensationalist content in newspapers with the volume of circulation, which is arguably a more objective assessment of readership interests. This is also demonstrated by Sumner et al. (2020) who found that online articles with greater fidelity to safe reporting practices were more likely to receive positive responses or to be reshared. It is imperative for the voice of lived experience to be included in the scientific discourse on the supply and demand of sensationalist suicide news reporting. This would be an important counterpoint to the risk of unsafe media narratives shaping public opinion on what is deemed socially acceptable in terms of suicide-news-reporting (Shanahan et al., 2011; Uzuegbunam and Udeze, 2013).

### Impact

Our findings were consistent with international findings (Collings and Kemp, 2010; Skehan et al., 2013; Sinyor et al., 2018; Gregory et al., 2020) where participants acknowledged both positive and negative impacts of encountering suicide news. Participants appreciated that suicide-related news promoted awareness on the topic and could help to advocate for suicide prevention (Skehan et al., 2013; Sinyor et al., 2018; Gregory et al., 2020). In our study, one participant acknowledged that encountering suicide news had indirectly improved their understanding related to suicide.

From the negative perspective, study participants who were bereaved experienced a re-traumatization and retriggering of their grief reactions, especially from encountering the details provided in a suicide story, whom they felt were unnecessary and unhelpful to readers (Gregory et al., 2020). Consistent with findings by Cheng et al. (2007), Niederkrotenthaler et al. (2012), and Fink et al. (2018), participants agreed that inappropriately reported celebrity suicides conferred a risk of copycat suicides (Niederkrotenthaler et al., 2012; Cheng et al., 2014; Fink et al., 2018). Participants in Cheng’s (2007) study described how their attention were drawn toward the suicide method and in turn ‘learned’ how to carry out a suicide from a media reporting of a celebrity suicide. Similarly, a PLE in our study described how a specific method of suicide kept ‘playing in (their) head’ upon encountering such information related to a celebrity suicide, in addition to the traumatic and intrusive nature of the experience. Other participants were negatively affected by suicide-related video footages and live tweets on social media; particularly by the negative comments that seemed to reinforce suicide acts rather than promote help-seeking. Similar worrying phenomena of ‘online suicide baiting’ have been described in Seko (2016), Brown et al. (2018), and Phillips and Mann (2019).

Our study also revealed firsthand, personal, and emotional experiences of how the news of suicide on the media affected MHPs, especially those with client suicide. Participants described the experience of being re-traumatized by distressing intrusive media descriptions or images of their clients. Such descriptions evoked feelings of anger and guilt, which impacted their professional duties. While similar traumatic reactions have been described in MHPs who have encountered client suicide, our findings extend this knowledge in that unsafe media content itself can serve as a significant trauma trigger (Chemtob et al., 1988; Wurst et al., 2013; Seguin et al., 2014; Gibbons et al., 2019).

With regards to the impact on media participants, our findings contrasted with those by Collings and Kemp (2010) and Armstrong et al. (2020), in that our media participants (especially those without lived experience) did not seem as personally affected (Collings and Kemp, 2010; Armstrong et al., 2020). This may be related to their “journalistic commitment to detachment, impartiality or professional distance” in order to remain professionally objective (Deuze, 2005; Kotišová, 2019; Armstrong et al., 2020). Barnes (2019) described how journalists are required to suppress, fake or enhance emotions during interactions as per media organization rules (Hopper and Huxford, 2015; Barnes, 2019). Another important reason may be that our media participants were not directly involved in covering suicide news which may involve cold-calling or interviewing the bereaved. For those who did, there are some anecdotal evidence that Malaysian journalists were emotionally affected as a result of covering suicide news (Yang, 2018; Lau, 2019).

### Safe Reporting Strategies

There is evidence in literature of the effectiveness of media guidelines in improving suicide-reporting practices and reducing suicide contagion (Bohanna and Wang, 2012). Our MHP participants posited that media professionals may be less motivated to abide by guidelines published that are not authored by one of their own profession. This is a view that has support in literature. Bohanna and Wang (2012)’s review indicates that the effectiveness of media guidelines will also require, amongst others; endorsement by the media community, consultation and collaboration – all in all, ‘media ownership.’ Studies in the United Kingdom (Norris et al., 2006) and New Zealand (Tully and Elsaka, 2004) have indicated that strong official advice, injunctions or restrictions from non-media industry sources on how to safely report suicide news is likely to be ‘resented, ignored or overlooked’ by journalists (Norris et al., 2006), a point that is reinforced recently by Duncan and Luce (2020). Even in the event of media industry self-regulation, the importance of ongoing collaboration and consultation with other suicide prevention stakeholders needs to be underscored (Tully and Elsaka, 2004; Norris et al., 2006). This perspective is echoed by the Canadian Psychiatric Association, which recommends ‘ongoing collaboration’ between media and mental health professionals that should acknowledge both the evidence base of the impacts of unsafe reporting and also the autonomy of journalists (Sinyor et al., 2018). In Austria, the involvement of the media industry in development, dissemination, and training processes for guidelines on suicide-reporting played a key role in changing reporting practices and reducing imitative suicide (Bohanna and Wang, 2012; Duncan and Luce, 2020). Similarly, in Australia, resources developed by suicide prevention non-profit institute with expertise on media and suicide prevention, *Mindframe*, in collaboration with media practitioners, were well received by journalists. In contrast, in China, media guidelines that were developed without the input of media practitioners, saw minimal ‘buy-in’, with reporting quality consequently remaining low (Tully and Elsaka, 2004; Fu and Yip, 2008; Bohanna and Wang, 2012). In New Zealand, prior to the coming into force of the Coroner’s Amendment Act 2016 which provisions involved media consultation, previous guidelines were criticized for lack of consultation during development and did not appear to have been used by journalists (Tully and Elsaka, 2004; Collings and Kemp, 2010; Bohanna and Wang, 2012; Duncan and Luce, 2020).

In terms of strategies for capacity-building within the media community, media professionals unanimously agreed that there is a need for more structured and specific training with regards to safe reporting for suicide prevention, beginning from journalism school and continuing throughout their professional career in the media industry. Duncan and Luce (2020) have created a free online Suicide Reporting Toolkit based on the *Responsible Suicide Reporting Model* which caters to building capacity across the board for journalist, editors, and educators. The toolkit aims to address the irregular uptake of guidelines by incorporating safe reporting in a practical manner by being grounded in news-work and journalistic storytelling. Importantly the toolkit’s real-world approach (narrative types, ethical rules, and standards of moderation) with regards to newsroom culture, especially tight deadlines and need for support for journalists covering suicide news, offers pragmatism in this landscape. Other potential points of intersectoral stakeholder collaboration in this area include curriculum-building at the education ministry level involving educators from the areas of both media and suicide prevention. Continuous professional education via workshops for media practitioners could also be considered as public-private partnerships between media organizations, and regulators such as the Malaysian Communications and Multimedia Commission within the Ministry of Communications and Multimedia.

### Intersectoral Collaboration

The common goals shared by advocates of both media and suicide prevention such as promotion of public good as one of the core values of journalistic ethics can serve as points of convergence and collaboration between stakeholders (Collings and Kemp, 2010; Cheng et al., 2014; Jenkin et al., 2020). This is to ensure that suicide news reporting is safe while still retaining authentic facts via careful phrasing, framing and contextualization of the media narrative. Similar to Skehan and colleagues, 2013 study, participants also highlighted the need for more prevention-focused reporting on suicide-related news, i.e., inclusion of relevant information on crisis help-seeking resources and provision of emotional support for the suicide-bereaved (Skehan et al., 2013). Anecdotal accounts have demonstrated that such a collaborative approach between mental health professionals and journalists can successfully influence editorial decisions toward safe suicide news reporting. Collaborating with media practitioners in improving the quality of safe suicide reporting could be one of the ways forward. The flexible nature of online/social media platforms enables changes to be made after publication. This may also facilitate real-time interventions as described by Martin, 2019, whereby unsafe content by a YouTuber was taken down following public outcry.

### Monitoring and Governance

The absence of specific guidance and monitoring of suicide-related safe reporting practices in the content code of the Malaysian Communications and Multimedia Content Code (Communications And Multimedia Content Forum Of Malaysia, 2004) were highlighted by the media. This finding is noteworthy, as the Code guides self-regulation by the media industry in compliance with the Communications and Multimedia Act 1998 (CMA 98) in Malaysia. Hence, revising and updating this content code appears to be a more strategic approach for implementation of media guidelines. However, compliance with the Code is dependent on voluntary participation of online, excluding print, media companies/websites registered in Malaysia. In 2020, a report and draft bill for the formation of a Malaysian media council encompassing print, broadcast and online media was proposed by the media industry to the Ministry of Communications and Multimedia (Protem Committee Malaysia Media Council, 2020). Elements of safe reporting on suicide-related content have been included in the draft bill with input from the mental health community. Such ongoing multi-lateral intersectoral engagement is a step toward broader stakeholder inclusivity. As previously indicated in our discussion, media industry ownership and adherence to self-regulation is likely to be higher compared to externally imposed health-centric guidelines that may be perceived as a threat to professional autonomy.

### Social Media

Focus group discussion participants in all groups consistently pointed out challenges posed by social media as an emerging source of news for readers in comparison to traditional media (i.e., print, radio, and television). In this context, issues arise in relation to the permanence and ease of access of online archives, and the added involvement of ‘citizen reporters’ as well as input by way of commenting and sharing, and even live streaming, by potentially any person who has access to social media platforms. This raises various issues. Unsafe suicide-related news content (including images) that is widely shared and interacted with may attain additional emphasis in terms of appearance on social media newsfeeds, giving such unsafe news more prominence even if it was not published with such intentions. While, unlike traditional media, it is possible for changes to be made after publication of a social media post to address any unsafe messaging, arguably such action would merely amount to mitigation rather than prevention. There is ample opportunity for further research to shed light on issues related to safe messaging in the context of social media networks.

Malaysia ranks the highest for mobile social media penetration in Southeast Asia with Facebook, a platform mentioned by our study participants; being one of the most popular (Kemp, 2020). At present, Facebook does not fall within the purview of existing national regulatory mechanisms. Self-regulatory mechanisms by Facebook such as artificial intelligence algorithms and engagement with suicide prevention experts (Facebook, 2021) are potential areas to build the evidence base for real-world safe messaging implementation.

### Other Interesting Findings From This Study

Our FGD provided a serendipitous avenue, similar to Balint’s group (Mahoney et al., 2013; Gerada, 2016) for MHPs to share and express their experiences related to losing their own clients to suicide. This was confirmed by an MHP participant who acknowledged feeling relieved after participating in the FGD for the opportunity to share their personal thoughts and feelings about their loss, and the knowledge that they were not alone, that their peers also had similar reactions.

During the course of the FGD, we encountered moments whereby content shared by some participants, especially those with lived experience, may have unintentionally triggered other participants. Those circumstances posed as challenging situations for the moderators who had to delicately address the situation and balance between the needs of the bereaved/affected to express and articulate their feelings while at the same time maintaining a safe environment to others present. Our study brings to surface the need for discussions between different stakeholders on how to communicate safely about suicide to be tempered by discretion and sensitivity to accommodate a spectrum of different nuances and diverse reactions (Dollah and Tandoc, 2020).

### Strengths and Limitations of the Study

To the best of our knowledge, our study is the first to explore perspectives among mental health professionals with regards to suicide reporting in the media, and our findings contribute to current knowledge related to the impact of client suicide on MHPs. Our findings also provide further insight on how media content with overly descriptive details related to suicide methods, as well as the interactive nature of suicide-related stories and/or news enabled by social media platforms, can further traumatize PLEs.

This study may also be the first to explore this subject in a multicultural population. In Malaysia, news platforms can be found in a diverse range of languages owing to the plural societal make-up of the country.

Our sampling methods and inclusion criteria resulted in the media-practitioner/student FGD participants being predominantly from English-language Malaysian media, although there was also some representation from Malay- and Chinese-language news portals. A limitation to be noted is that the media practitioners/students in our sample had not been directly involved in covering news related to suicides. Hence, findings from our study have some limitations in terms of generalizability to non-English media. Another study limitation is the lack of information on the representation of tabloid versus high-quality media.

It should also be noted that the sample of mental health professionals in this study consisted only of psychiatrists from public healthcare institutions. It would be useful for further studies to explore the perspectives of other mental health practitioners working in suicide prevention, such as private sector psychiatrists, clinical psychologists, counselors, social workers, public health professionals, and mental health advocates.

## Conclusion

From our study, there seemed to be a low level of awareness with regards to existing local media guidelines on safe reporting of suicide-related content amongst the media, mental health professionals and people with lived experience of suicidal behavior. In addition, reporting unsafe media content can be traumatizing for media users with lived experience, including mental health practitioners who have been impacted by client suicide. Furthermore, our findings highlighted the need for postvention support for affected individuals, which is especially lacking for mental health professionals. Given the prevalence of unsafe reporting on social media platforms, there is a need for media guidelines to address this emerging area. Finally, despite the differing needs and experiences of stakeholder groups, we have found shared commonality and agreements on the need for safe reporting. Therefore, parties involved in suicide reporting can capitalize on shared values and adapt dynamically to the perspectives of, and impacts on, diverse stakeholders.

## Data Availability Statement

The original contributions generated for this study are included in the article/Supplementary Material, further inquiries can be directed to the corresponding author.

## Ethics Statement

The studies involving human participants were reviewed and approved by Universiti Tunku Abdul Rahman Scientific and Ethical Review Committee (SERC). The patients/participants provided their written informed consent to participate in this study.

## Author Contributions

YN, KP, RP, WC, LC, JL, and SB: conceptualization study design. YN, KP, RP, WC, JTL, KL, and KT: focus group discussion. YN, RP, WC, JTL, and KL: transcription. YN, KP, RP, WC, and LC: data analysis. YN, KP, RP, WC, JL, LC, and KT: drafting of manuscript. YN, KP, RP, WC, LC, JTL, JL, SB, KL, and KT: review and final approval of manuscript. All authors contributed to the article and approved the submitted version.

## Conflict of Interest

The authors declare that the research was conducted in the absence of any commercial or financial relationships that could be construed as a potential conflict of interest.
