# Supplementary material for: Malaysian Stakeholder Perspectives on Suicide-Related Reporting: Findings From Focus Group Discussions
Source: Front Psychol. 2021 May 17;12:673287. doi: 10.3389/fpsyg.2021.673287 (PMC8165722; doi:10.3389/fpsyg.2021.673287)
Supplement: Supplementary file 1 [file Table_1.docx]

**Supplementary Table**

| **Media Reporting of Suicide News** | | |
| --- | --- | --- |
| **Major Themes (3)** | | |
| **Unsafe reporting**  Communication regarding suicide related news content and context resulting in negative consequences on to readers either traumatic and/or triggering contagion.  **Participant consensus:**  Media – all 8  MHP – all 7  PLE – all 6 | **Impact**  This theme explores the positive and negative effects of media reporting of suicide news in suicide prevention both in personal and professional aspects. | **Safe reporting**  Suicide news coverage that is helpful in suicide prevention. This theme explores qualities of safe reporting along personal, systemic and economic factors including challenges and mitigation factors to improve reporting quality.  (There is significant overlap in terms of the influencing factors and challenges for safe reporting with the factors/ reasons related to unsafe reporting. As such, the participant consensus for those subtheme items is not shown.) |
| **Subthemes:** | | |
| **Forms of unsafe reporting**   - Content - Unnecessary details (methods/ graphics/ pictures) - Negative content – provocative, sensationalist and unsupportive language - Lack of help-seeking resources - Framing of content - Judgmental, blame-inducing - Inaccurate portrayal - Romanticised suicide as desirable: painless, peaceful, coping solution - Decedent idolisation - Help-seeking as difficult - Accessibility   - Online for wider reach   - Permanently available at own convenience - Prominence   - Front page, Headline   - Repeated coverage of same report | **Negative effects of suicide reporting**   - Triggers traumatic responses   - - - Failure feelings for mental health professionals       - Reigniting traumatic memories of own crises in people with lived experience       - Intrusive images of graphic material seen on media - Contagion risk effects - Intrusive images/thoughts of methods - Propagates myths & stigma related to mental illness and suicide   **Participant consensus:**  Media – all 8  MHP – 6 were personally affected, 1 gave a general statement  PLE – all 6 | **Concept Definition**  Content and emphasis  This subtheme explores the qualities of a safe suicide news report:   - - Includes     - Fidelity towards media guidelines   **Participant consensus:**  Media – 7/8  MHP – 6/7  PLE – 2/6   - - - Prioritises & focuses on safety & positive messages   **Participant consensus:**  Media – 5/8  MHP – 4/7  PLE – 5/6   - - - Provides help-seeking resources, with     - Larger emphasis on help-seeking resources, placed strategically   **Participant consensus:**  Media – 4/8  MHP – all 7  PLE – 3/6   - - - Considerations on the impact on media users including       - Trigger warnings where needed       - Accurate information   **Participant consensus:**  Media – 6/8  MHP – 5/7  PLE – 4/6   - - - Newsworthiness   - Excludes harmful components such as     - Sensationalism     - Speculation     - Unnecessary details/ graphic descriptions   **Participant consensus:**  Media – 5/8 (participants were divided with regards of the level of details)  MHP – all 7  PLE – 5/6 |
| **Reasons for unsafe reporting**   - Inadequate knowledge, awareness & guidance   **Participant consensus:**  Media – all 8  MHP – all 7  PLE – 2/6   - No monitoring/ governance   **Participant consensus:**  Media – 6/8  MHP – 5/7  PLE –   - Job factors such as business sustainability (perceived customer demand and newsworthiness), perceived duty to report the truth, final decision being dependent on editorial discretion.   **Participant consensus:**  Media – 5/8  MHP – 4/7  PLE – 4/6   - Information source with unsafe content   **Participant consensus:**  Media – 2/8  MHP – 3/7  PLE –   - Lack of empathy and accountability   **Participant consensus:**  Media – 5/8  MHP – 5/7  PLE – 1/6   - Popularity-seeking   **Participant consensus:**  Media – 5/8  MHP – 1/7  PLE – | **Positive effects of suicide reporting**   - Raises curiosity & awareness - Facilitates discussion on suicide prevention - Evokes empathy towards people with lived experience - Promotes a sense of responsibility & desire to help - Improves understanding of suicide and its prevention   **Participant consensus:**  Media – 7/8  MHP – all 7  PLE – all 6 | **Influencing factors towards safe reporting**   - Level of knowledge & awareness related to suicide prevention - Presence of empathy and accountability - Job factors (eg. time constraints, newsworthiness, business sustainability, editorial discretion) - Information source   Monitoring & liability implications   - Contact with people with lived experience   **Participant consensus:**  Media – 6/8  MHP – 3/7  PLE – not mentioned |
|  |  | **Challenges in safe reporting**   - Difficulty deciding content-safety   **Participant consensus:**  Media – 4/8  MHP – 4/7  PLE – 1/7   - Engaging media/ editor buy-in   **Participant consensus:**  Media – 4/8  MHP – 4/7  PLE – 3/6   - Poor awareness/ knowledge (including those related to media guidelines) - Lack of guidance and training - Difficulty in monitoring especially social media, lack of governance - Cultivating interest and empathy - Information sources with unsafe content - Balancing job/ business sustainability |
|  |  | **Strategies in improving safe reporting**   - Education & training for all stakeholders especially on safe reporting.   **Participant consensus:**  Media – all 8  MHP – all 7  PLE – 5/6   - Stakeholder collaboration to improve engagement. Measures include: - providing incentives to empower safe reporting practices.   **Participant consensus:**  Media – 4/8  MHP – all 7  PLE – 4/7   - Engaging and collaborating with the media   **Participant consensus:**  Media – 6/8  MHP – all 7  PLE – 3/6   - Media guideline revision, engagement (involving social media) to improve uptake   **Participant consensus:**  Media – 7/8  MHP – 6/7  PLE – 3/6   - Governance & surveillance including the role of legislation   **Participant consensus:**  Media – 4/8; 2/8 disagreed on enforcement  MHP – 6/7  PLE – 2/6 (towards governance and empowerment rather than enforcement). |
